# Supplementary figures and images for: Female vulnerability to the effects of smoking on health outcomes in older people
Source: PLoS One. 2020 Jun 4;15(6):e0234015. doi: 10.1371/journal.pone.0234015 (PMC7272024; doi:10.1371/journal.pone.0234015)

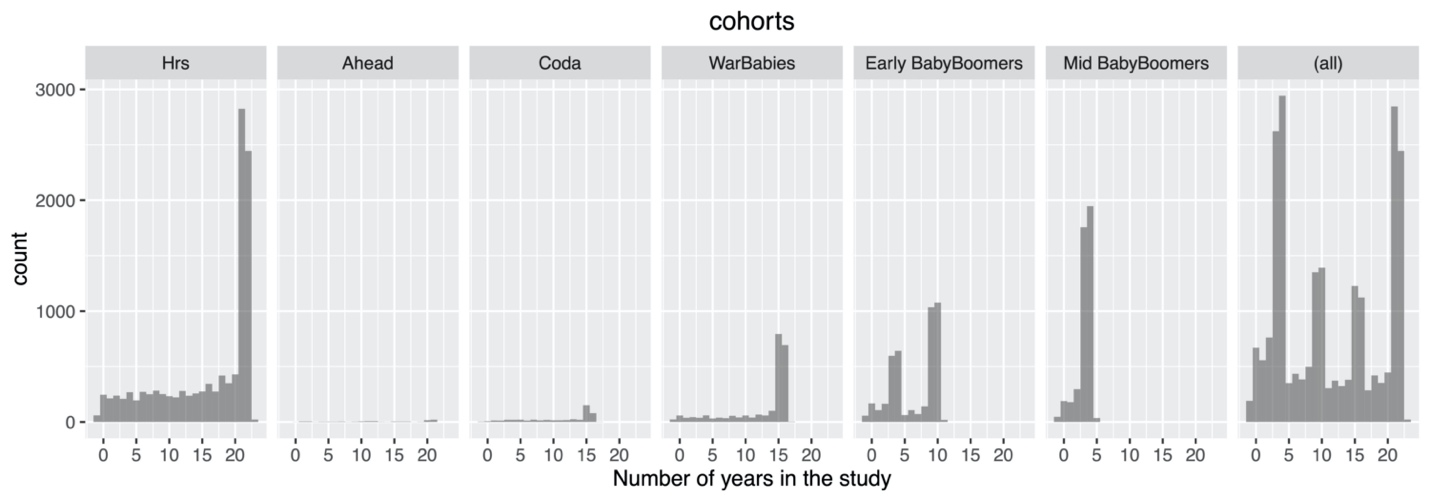


Figure S1. Histogram of number of years of in different cohorts enrolled in HRS.

Supplement: S1 Fig — (DOCX) [file pone.0234015.s001.docx]

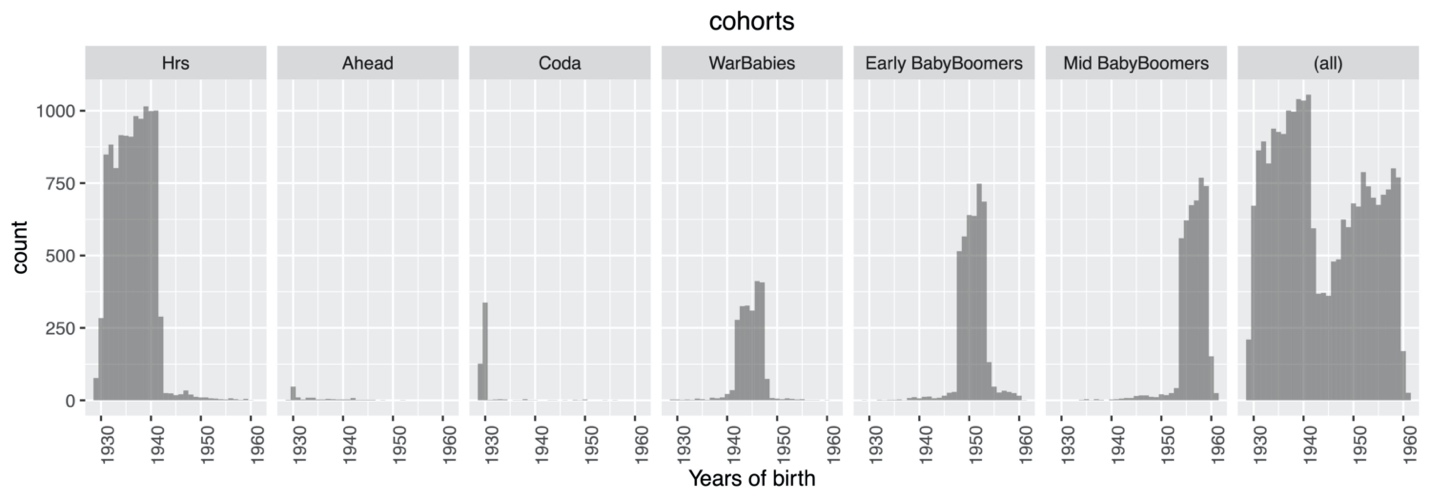
 Figure S2. Histogram of year of birth of different cohorts enrolled in HRS.

Supplement: S2 Fig — (DOCX) [file pone.0234015.s002.docx]

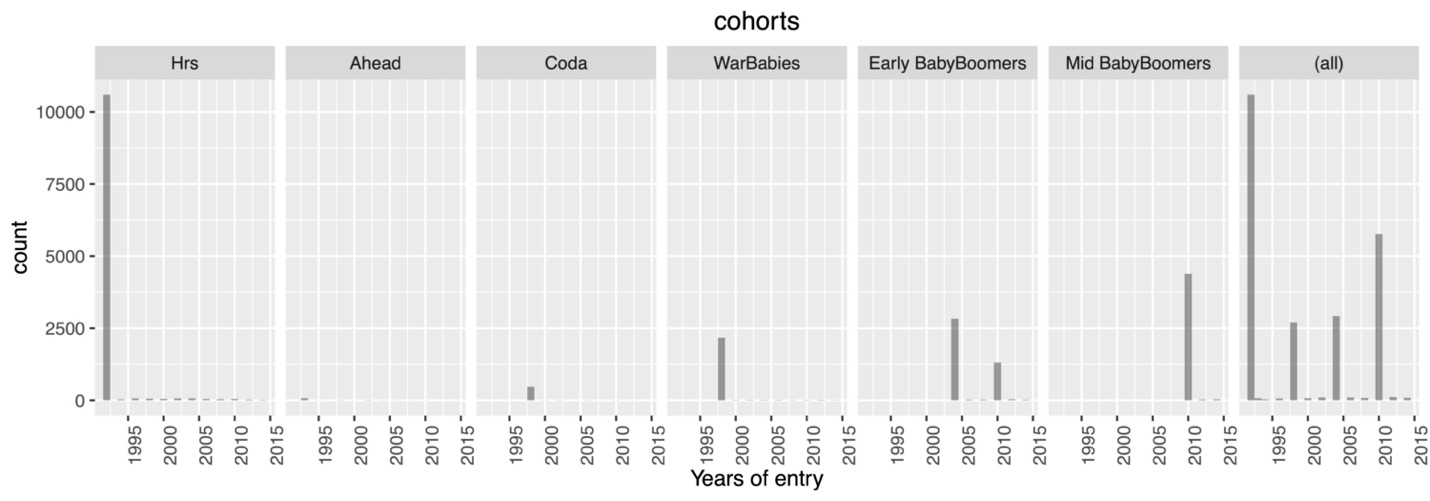
 Figure S3. Histogram of year of recruitment for each cohort in HRS.

Supplement: S3 Fig — (DOCX) [file pone.0234015.s003.docx]
