# Supplementary material for: Female vulnerability to the effects of smoking on health outcomes in older people
Source: PLoS One. 2020 Jun 4;15(6):e0234015. doi: 10.1371/journal.pone.0234015 (PMC7272024; doi:10.1371/journal.pone.0234015)
Supplement: S4 Fig — The significance of difference between current and former smokers was assessed by t-test. * p<0.05. (DOCX) [file pone.0234015.s004.docx]

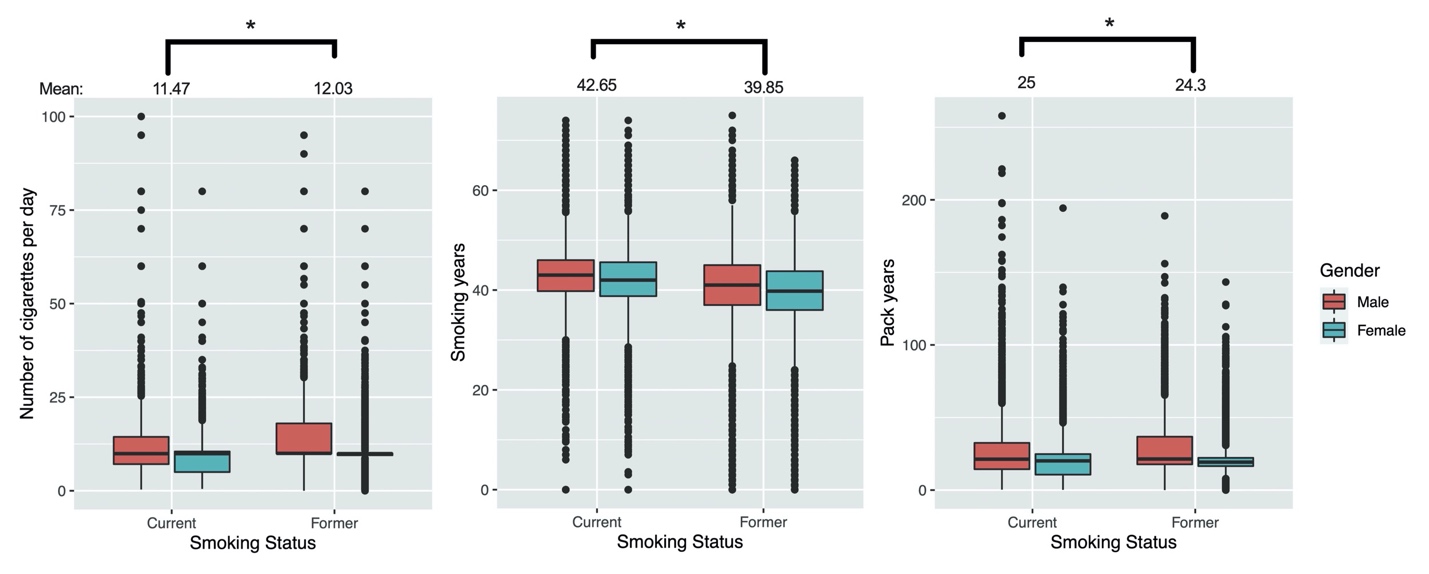
 Figure S4. Box plot of average number of cigarettes per day, smoking years, and pack years of 17,399 men and women current or former smokers in HRS. The significance of difference between current and former smokers was assessed by t-test. * p<0.05.
