# Supplementary material for: Female vulnerability to the effects of smoking on health outcomes in older people
Source: PLoS One. 2020 Jun 4;15(6):e0234015. doi: 10.1371/journal.pone.0234015 (PMC7272024; doi:10.1371/journal.pone.0234015)
Supplement: S5 Fig — In total, 12585 respondents had complete data, and 10123 respondents had at least one missing variable that had to be imputed (e.g. age of start smoking, age of quitting smoking, average daily cigarettes smoked). (DOCX) [file pone.0234015.s005.docx]

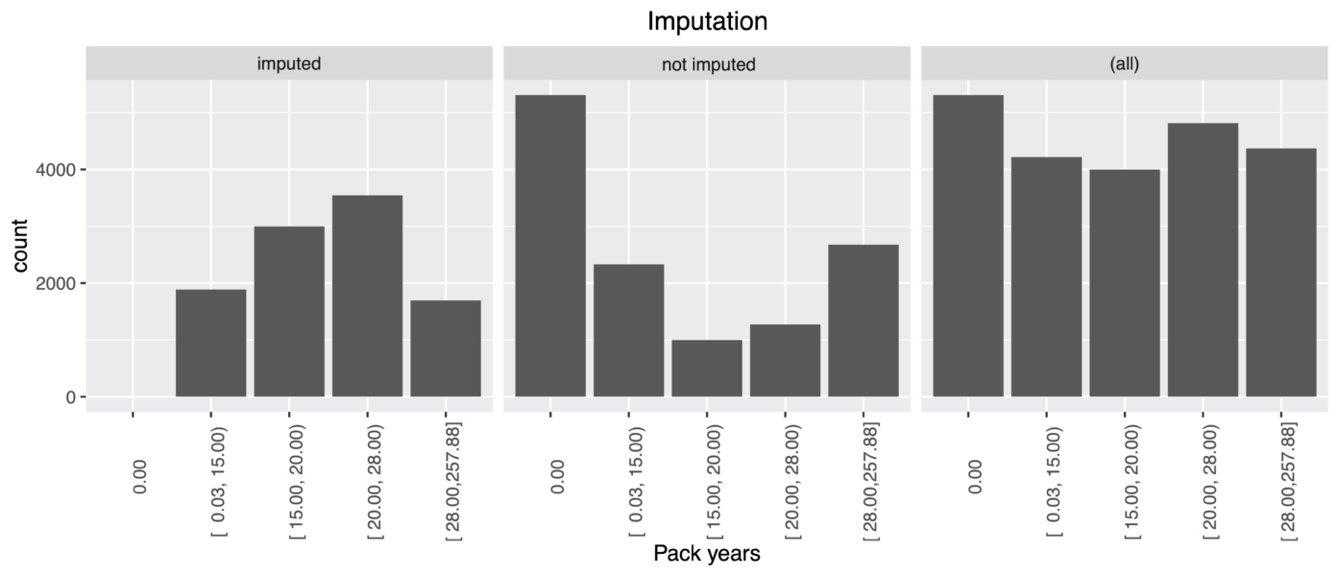


Figure S5. Histogram of the number of individuals with imputations used in creating the pack year variable. In total, 12585 respondents had complete data, and 10123 respondents had at least one missing variable that had to be imputed (e.g. age of start smoking, age of quitting smoking, average daily cigarettes smoked).
