# Supplementary material for: Female vulnerability to the effects of smoking on health outcomes in older people
Source: PLoS One. 2020 Jun 4;15(6):e0234015. doi: 10.1371/journal.pone.0234015 (PMC7272024; doi:10.1371/journal.pone.0234015)
Supplement: S1 Table — (DOCX) [file pone.0234015.s006.docx]

Table S1. Demographic characteristics of the HRS sample, 1992-2014

|  |  | Men | Women |
| --- | --- | --- | --- |
| N |  | 10945 (48.2%) | 11763 (51.8%) |
| Age, years (range) |  | 66.58 (50-85) | 66 (50-85) |
| Ethnicity |  |  |  |
|  | White/Caucasian | 7186 (65.7%) | 7211 (61.3%) |
|  | African American | 1983 (18.1%) | 2636 (22.4%) |
|  | Hispanic | 1409 (12.9%) | 1546 (13.1%) |
|  | Other | 367 (3.4%) | 370 (3.1%) |
| Smoking |  |  |  |
|  | Never smoker | 1458 (13.3%) | 1901 (16.2%) |
|  | Passive smoker | 695 (6.3%) | 1255 (10.7%) |
|  | Ever smoker (Current and former) | 8792 (80.3%) | 8607 (73.2%) |
| Pack years | Category Range |  |  |
|  | None (includes passive smokers) | 2153 (19.7%) | 3156 (26.8%) |
|  | Low (0.03-15.0) | 1904 (17.4%) | 2312 (19.7%) |
|  | Medium (15.1-20.0) | 1640 (15.0%) | 2358 (20.0%) |
|  | High (20.1-28.0) | 2438 (22.3%) | 2377 (20.2%) |
|  | Very high (28.1-258.0) | 2810 (25.7%) | 1560 (13.3%) |
| Years since quitting |  |  |  |
|  | Non-smoker (includes passive smokers) | 2151 (19.7%) | 3154 (26.8%) |
|  | Current smokers | 3528 (32.2%) | 3032 (25.8%) |
|  | <5 | 1294 (11.8%) | 1121 (9.5%) |
|  | 5-15 | 1366 (12.5%) | 1193 (10.1) |
|  | >15 | 2606 (23.8%) | 3263 (27.7%) |
| Disease prevalence |  |  |  |
|  | Lung disorders | 642 (5.9%) | 826 (7.0%) |
|  | Heart disease | 2848 (26.0%) | 2603 (22.1%) |
|  | Stroke | 1115 (10.2%) | 1089 (9.3%) |
|  | Cancer | 1952 (17.8%) | 2116 (18.0%) |

Note: reported percentages for each variable are within gender. Pack-years quartiles were calculated among smokers only.
