# Supplementary material for: Female vulnerability to the effects of smoking on health outcomes in older people
Source: PLoS One. 2020 Jun 4;15(6):e0234015. doi: 10.1371/journal.pone.0234015 (PMC7272024; doi:10.1371/journal.pone.0234015)
Supplement: S2 Table — This subpopulation showed a similar pattern but stronger sex-smoking interaction for the age of death, heart disease, and cancer. Thus, the main text reported the results of the whole population. (DOCX) [file pone.0234015.s007.docx]

Table S2. Hazard ratios of age of death, and age of onset of lung disorders, heart disease, and stroke according to ever smoking and the interaction with gender in data with no imputation. This subpopulation showed a similar pattern but stronger sex-smoking interaction for the age of death, heart disease, and cancer. Thus, the main text reported the results of the whole population.

| variable | level HR (95%CI) | **Age of death** | **Lung disorders** | **Heart disease** | **Stroke** | **Cancer** |
| --- | --- | --- | --- | --- | --- | --- |
| gender | Men (ref) |  |  |  |  |  |
|  | Women | **0.58 (0.47 ,0.72)***** | **2.85 (1.75 ,4.63)***** | **0.82 (0.71 ,0.95)**** | 0.8 (0.63 ,1.02) | 1.13 (0.96 , 1.33) |
| ethnicity | White/Caucasian (ref) |  |  |  |  |  |
|  | African American | **1.52 (1.35 ,1.71)***** | 0.86 (0.71 ,1.03) | 1 (0.91 ,1.10) | **2.13 (1.86 ,2.44)***** | **0.85 (0.76 , 0.96)**** |
|  | Hispanic | 0.97 (0.81 ,1.15) | 0.78 (0.61 ,1.00) | **0.78 (0.68 ,0.89)***** | **1.42 (1.18 ,1.72)***** | **0.77 (0.66 , 0.89)**** |
|  | other | 0.93 (0.66 ,1.31) | 1.3 (0.90 ,1.89) | 1.03 (0.83 ,1.29) | 1.31 (0.92 ,1.87) | **0.73 (0.54 , 0.97)*** |
| Pack years | Non-smokers (ref) |  |  |  |  |  |
|  | Low | **0.75 (0.60 ,0.94)*** | **3.74 (2.25 ,6.23)***** | 1.09 (0.92 ,1.29) | **1.29 (1.00 ,1.67)*** | 1.2 (0.98 , 1.45) |
|  | Medium | 0.83 (0.63 ,1.08) | **4.56 (2.64 ,7.87)***** | 1.2 (0.98 ,1.46) | 1.15 (0.83 ,1.59) | **1.32 (1.05 , 1.65)*** |
|  | High | **0.65 (0.51 ,0.83)***** | **5.9 (3.60 ,9.67)***** | **1.4 (1.18 ,1.66)***** | 1.23 (0.93 ,1.62) | 1.05 (0.85 , 1.29) |
|  | Very high | 1.06 (0.88 ,1.28) | **8.13(5.15 ,12.82)***** | **1.46 (1.27 ,1.67)***** | **1.48 (1.18 ,1.85)**** | **1.21 (1.03 , 1.42)*** |
| Women x Smoking interaction | Low | 1.17 (0.86 ,1.59) | **0.41 (0.23 ,0.74)**** | 1.2 (0.96 ,1.49) | 0.84 (0.59 ,1.18) | 0.78 (0.61 , 1.01) |
|  | Medium | **1.61 (1.11 ,2.34)*** | 0.6 (0.32 ,1.14) | **1.46 (1.11 ,1.91)**** | **1.66 (1.08 ,2.54)*** | **0.59 (0.43 , 0.83)**** |
|  | High | **1.94 (1.39 ,2.70)***** | **0.51 (0.28 ,0.90)**** | 0.92 (0.72 ,1.17) | 1.3 (0.89 ,1.92) | 0.93 (0.70 , 1.24) |
|  | Very high | **1.49 (1.14 ,1.93)**** | **0.52 (0.31 ,0.87)**** | 1.09 (0.90 ,1.32) | 1.36 (0.99 ,1.85) | 0.91 (0.72 , 1.13) |
| Total N |  | 12585 | 11889 | 12585 | 12577 | 12569 |

Note: * p < 0.05, ** p < 0.01, *** p < 0.001
