# Supplementary material for: Female vulnerability to the effects of smoking on health outcomes in older people
Source: PLoS One. 2020 Jun 4;15(6):e0234015. doi: 10.1371/journal.pone.0234015 (PMC7272024; doi:10.1371/journal.pone.0234015)
Supplement: S4 Table — Confounders: Years of education, Cohort, Ethnicity. (DOCX) [file pone.0234015.s009.docx]

Table S4. Hazard ratios of age of death, and age of onset of lung disorders, heart disease, and stroke according to ever smoking and the interaction with gender in data with no imputation. Confounders: Years of education, Cohort, Ethnicity.

| variable | level HR (95%CI) | **Age of death** | **Lung disorders** | **Heart disease** | **Stroke** | **Cancer** |
| --- | --- | --- | --- | --- | --- | --- |
| gender | Men (ref) |  |  |  |  |  |
|  | Women | **0.66 (0.53 – 0.81)***** | **3.06 (1.88 – 4.98)***** | **0.86 (0.74 – 1.00)*** | 0.82 (0.65 – 1.05) | **1.23 (1.04 – 1.45)*** |
| Years of education |  | **0.95 (0.95 – 0.96)***** | **0.95 (0.93 – 0.96)***** | **0.99 (0.98 – 0.99)***** | **0.98 (0.97 – 0.99)**** | 1 (1.00 – 1.01) |
| Cohort | HRS |  |  |  |  |  |
|  | AHEAD | 1.07 (0.79 – 1.45) | 0.63 (0.30 – 1.34) | 0.95 (0.69 – 1.31) | 0.79 (0.44 – 1.39) | **0.55 (0.35 – 0.88)*** |
|  | CODA | **0.83 (0.71 – 0.97)*** | **0.7 (0.50 – 0.97)*** | **0.74 (0.63 – 0.88)***** | 1.07 (0.85 – 1.35) | **0.76 (0.63 – 0.92)**** |
|  | WarBabies | 1.09 (0.97 – 1.23) | **1.72 (1.45 – 2.04)***** | **1.14 (1.03 – 1.25)**** | **1.24 (1.06 – 1.45)**** | **1.22 (1.10 – 1.37)***** |
|  | Early BabyBoomers | 1.06 (0.93 – 1.22) | **1.46 (1.19 – 1.78)***** | 1.08 (0.98 – 1.18) | **1.34 (1.16 – 1.55)***** | **1.19 (1.06 – 1.33)**** |
|  | Mid BabyBoomers | 1.15 (0.93 – 1.42) | **1.69 (1.26 – 2.26)***** | **1.41 (1.27 – 1.57)***** | **1.44 (1.21 – 1.72)***** | **1.48 (1.31 – 1.68)***** |
| ethnicity | White/Caucasian (ref) |  |  |  |  |  |
|  | African American | **1.5 (1.40 – 1.62)***** | **0.82 (0.70 – 0.95)**** | 1 (0.93 – 1.07) | **1.95 (1.76 – 2.16)***** | **0.83 (0.76 – 0.91)***** |
|  | Hispanic | **0.84 (0.74 – 0.94)**** | **0.6 (0.48 – 0.74)***** | **0.74 (0.67 – 0.82)***** | 1.2 (1.03 – 1.39)* | **0.72 (0.64 – 0.81)***** |
|  | other | 1.01 (0.82 – 1.23) | 1.23 (0.91 – 1.67) | 0.99 (0.84 – 1.17) | 1.12 (0.85 – 1.48) | **0.7 (0.57 – 0.87)**** |
| Pack years | Non-smokers (ref) |  |  |  |  |  |
|  | Low | **1.35 (1.12 – 1.65)**** | **3.49 (2.12 – 5.74)***** | 1.15 (0.99 – 1.34) | **1.33 (1.05 – 1.68)*** | **1.29 (1.08 – 1.53)**** |
|  | Medium | **1.44 (1.18 – 1.75)***** | **3.81 (2.32 – 6.26)***** | **1.32 (1.14 – 1.54)***** | **1.43 (1.12 – 1.82)**** | **1.4 (1.18 – 1.67)***** |
|  | High | **1.38 (1.15 – 1.66)***** | **3.86 (2.41 – 6.21)***** | **1.41 (1.23 – 1.61)***** | **1.38 (1.11 – 1.73)**** | **1.26 (1.07 – 1.49)**** |
|  | Very high | **2.14 (1.79 – 2.56)***** | **8.11 (5.12 – 12.84)***** | **1.62 (1.42 – 1.85)***** | **1.71 (1.38 – 2.13)***** | **1.43 (1.22 – 1.68)***** |
| Women x Smoking interaction | Low | 1.1 (0.84 – 1.43) | **0.42 (0.24 – 0.75)**** | 1.15 (0.94 – 1.40) | 0.95 (0.70 – 1.31) | 0.82 (0.66 – 1.03) |
|  | Medium | 0.99 (0.77 – 1.28) | **0.4 (0.23 – 0.69)**** | 0.92 (0.76 – 1.11) | 1.01 (0.74 – 1.38) | **0.75 (0.60 – 0.93)**** |
|  | High | 1.16 (0.91 – 1.48) | **0.43 (0.26 – 0.74)**** | 0.87 (0.73 – 1.04) | 1.11 (0.83 – 1.49) | 0.82 (0.67 – 1.01) |
|  | Very high | **1.28 (1.00 – 1.62)*** | **0.53 (0.32 – 0.88)*** | 1.02 (0.85 – 1.23) | **1.35 (1.01 – 1.80)*** | 0.91 (0.74 – 1.12) |
| Total N |  | 22708 | 21486 | 22708 | 22695 | 22689 |

Note: * p < 0.05, ** p < 0.01, *** p < 0.001
