# Supplementary material for: Female vulnerability to the effects of smoking on health outcomes in older people
Source: PLoS One. 2020 Jun 4;15(6):e0234015. doi: 10.1371/journal.pone.0234015 (PMC7272024; doi:10.1371/journal.pone.0234015)
Supplement: S5 Table — (DOCX) [file pone.0234015.s010.docx]

Table S5. Hazard ratios of age of death, and age of onset of lung disorders, heart disease, stroke and cancer according to years since quitting smoking and the interaction with sex.

| variable | level HR (95%CI) | **Age of death** | **Lung disorders** | **Heart disease** | **Stroke** | **Cancer** |
| --- | --- | --- | --- | --- | --- | --- |
| Sex | Men (ref) |  |  |  |  |  |
|  | Women | **0.61 (0.49 , 0.75)***** | **2.83 (1.74 , 4.59)***** | **0.82 (0.71 , 0.95)**** | 0.8 (0.63 , 1.02) | 1.13 (0.96 , 1.33) |
| ethnicity | White/Caucasian (ref) |  |  |  |  |  |
|  | African American | **1.31 (1.22 , 1.40)***** | **0.74 (0.64 , 0.86)***** | 0.99 (0.92 , 1.06) | **1.87 (1.70 , 2.06)***** | **0.83 (0.76 , 0.90)***** |
|  | Hispanic | 0.98 (0.88 , 1.08) | **0.67 (0.55 , 0.82)***** | **0.76 (0.70 , 0.84)***** | **1.26 (1.10 , 1.44)***** | **0.73 (0.65 , 0.81)***** |
|  | other | 0.91 (0.74 , 1.11) | 1.13 (0.83 , 1.53) | 0.98 (0.83 , 1.16) | 1.09 (0.82 , 1.43) | **0.71 (0.57 , 0.88)**** |
| Years of quitting | Non-smokers (ref) |  |  |  |  |  |
|  | Current smoker | **1.29 (1.07 , 1.55)**** | **4.3 (2.69 , 6.86)***** | **1.29 (1.13 , 1.47)***** | **1.32 (1.06 , 1.64)*** | 1.14 (0.97 , 1.34) |
|  | <5 | **6.26 (5.27 , 7.44)***** | **8.24 (5.12 , 13.24)***** | **1.5 (1.29 , 1.75)***** | **2 (1.59 , 2.53)***** | **1.55 (1.30 , 1.85)***** |
|  | 5-15 | **3.43 (2.89 , 4.08)***** | **6.59 (4.13 , 10.49)***** | **1.47 (1.27 , 1.69)***** | **1.77 (1.42 , 2.22)***** | **1.2 (1.01 , 1.42)*** |
|  | >15 | **0.48 (0.40 , 0.57)***** | **2.67 (1.68 , 4.23)***** | **1.22 (1.08 , 1.39)**** | 0.89 (0.71 , 1.10) | 1.08 (0.93 , 1.25) |
| Women x Smoking quit interaction | Current smoker | 1.14 (0.87 , 1.48) | **0.55 (0.32 , 0.94)*** | 1.06 (0.88 , 1.27) | 1.23 (0.92 , 1.65) | 1.09 (0.88 , 1.34) |
|  | <5 | **1.33 (1.05 , 1.69)*** | **0.51 (0.30 , 0.88)*** | 1.01 (0.81 , 1.26) | 1.09 (0.79 , 1.52) | 0.98 (0.77 , 1.24) |
|  | 5-15 | **1.41 (1.11 , 1.79)**** | **0.37 (0.22 , 0.63)***** | 1.05 (0.86 , 1.28) | 1.08 (0.80 , 1.48) | 0.99 (0.79 , 1.25) |
|  | >15 | 1.18 (0.92 , 1.52) | **0.39 (0.23 , 0.65)***** | 0.91 (0.77 , 1.08) | 1.08 (0.82 , 1.44) | **0.71 (0.59 , 0.87)**** |
| Total N |  | 22708 | 21486 | 22708 | 22695 | 22689 |

Note: * p < 0.05, ** p < 0.01, *** p < 0.001
