# Supplementary material for: Female vulnerability to the effects of smoking on health outcomes in older people
Source: PLoS One. 2020 Jun 4;15(6):e0234015. doi: 10.1371/journal.pone.0234015 (PMC7272024; doi:10.1371/journal.pone.0234015)
Supplement: Attachment — Submitted filename: Respond to reviewers, May 14.docx [file pone.0234015.s012.docx]

Review Comments to the Author

Please use the space provided to explain your answers to the questions above. You may also include additional comments for the author, including concerns about dual publication, research ethics, or publication ethics. (Please upload your review as an attachment if it exceeds 20,000 characters)

**Reviewer #1:**

Reviewer #1: I thank the authors for the thorough revision of the manuscript and conducting several additional analyses. I just have one further comment regarding the analyses on smoking cessation. Investigating sex-specific effects of smoking cessation on different health outcomes might be beyond the scope of this manuscript and its aims. As in the first version of the manuscript the exact definition of “active smokers” (now indicated as “ever smokers”) was not entirely clear and the assessment of lifetime pack-years smoked differs by smoking status (average number of cigarettes per day vs. maximum number of cigarettes), sensitivity analyses investigating whether the effects of smoking on health outcomes differ by smoking status might have been reasonable. Therefore, ever smokers could have been separated into former and never smokers (taking lifetime pack-years into account) independent of years since smoking cessation. Table 3 in the manuscript shows some quite high hazard ratios with broad confidence intervals. This might be due to the small sample sizes, esp. for women who quit smoking <5 or 5-15 years ago and who have lung disorders. Thus, these results should be interpreted with caution. I would suggest to move these results to the supplement and not put too much emphasis on this kind of analyses. Rather, I would just add this as kind of sensitivity analyses (as was done e.g. for the comparison of imputed vs. non-imputed pack-years data) to see whether the effects change and just mention this briefly. Nevertheless, I would like to thank the authors for all their efforts.

Answer: Thank you for this comment. As suggested, table 3 was moved to the supplement. We also softened the language about the conclusions on sex differences in the benefits of smoking cessation.
